# Fabrication and Characterization of Bio-Nanocomposites Based on Halloysite-Encapsulating Grapefruit Seed Oil in a Pectin Matrix as a Novel Bio-Coating for Strawberry Protection

**DOI:** 10.3390/nano12081265

**Published:** 2022-04-08

**Authors:** Gianluca Viscusi, Elena Lamberti, Francesca D’Amico, Loredana Tammaro, Giuliana Gorrasi

**Affiliations:** 1 Department of Industrial Engineering, University of Salerno, Via Giovanni Paolo II, 132, 84084 Fisciano, SA, Italy; gviscusi@unisa.it (G.V.); ellamberti@unisa.it (E.L.); f.damico15@studenti.unisa.it (F.D.); 2 Nanomaterials and Devices Laboratory (SSPT-PROMAS-NANO), ENEA—Italian National Agency for New Technologies, Energy and Sustainable Economic Development, Piazzale E. Fermi, 1, 80055 Portici, NA, Italy; loredana.tammaro@enea.it

**Keywords:** active coating, biocomposites, grapefruit seed oil, halloysite, nano-hybrid, pectin

## Abstract

In the framework of designing a novel bio-coating for the preservation of fresh fruits, this paper reports the design, preparation, and characterization of novel bio-nanocomposites based on pectin loaded with grapefruit seed oil (GO), a natural compound with antimicrobial properties, encapsulated into halloysite nanotubes (HNTs). The vacuum-based methodology was used for the encapsulation of the oil into the hollow area of the nanotubes, obtaining nano-hybrids (HNT-GO) with oil concentrations equal to 20, 30, and 50 wt%. Physical properties (thermal, mechanical, barrier, optical) were analyzed. Thermal properties were not significantly (*p* < 0.05) affected by the filler, while an improvement in mechanical performance (increase in elastic modulus, stress at breaking, and deformation at breaking up to 200%, 48%, and 39%, respectively, compared to pure pectin film) and barrier properties (increase in water permeability up to 480% with respect to pure pectin film) was observed. A slight increase in opacity was detected without significantly compromising the transparency of the films. The release of linoleic acid, the main component of GO, was followed for 21 days and was correlated with the amount of the hybrid filler, demonstrating the possibility of tailoring the release kinetic of active molecules. In order to evaluate the effectiveness of the prepared bio-composites as an active coating, fresh strawberries were coated and compared to uncoated fruit. Qualitative results showed that the fabricated novel bio-coating efficiently extended the preservation of fresh fruit.

## 1. Introduction

Active, intelligent, and edible packaging aiming to reduce the cost and the amount of traditional material-based packaging as well as control gases, moisture, and lipid migration and supporting additives and nutrients is attracting great interest in both basic and applied research [1,2,3].

Polysaccharides, proteins, and lipids are the most usable materials for the fabrication of edible films that must increase the shelf-life of the packaged food, limiting the contamination of the environment [4]. The film-forming ability of several natural polymers (i.e., cellulose [5], chitosan [6], starch [7], pectin [8], alginate [9], carrageenan [10], pullulan [11], and kefiran [12]) has been studied. Often, the addition of plasticizers is required in order to obtain protein- and polysaccharide-based films, otherwise films are characterized by brittleness due to interactions between polymer chains [13]. Their role is to reduce the cohesion within the film network by weakening the intermolecular forces between adjacent polymer chains [8,14], affecting barrier and mechanical properties, the tension of deformation, hardness, density, and viscosity [15]. Among the best polysaccharides to be used as coating for food protection, pectins have been demonstrated to be excellent materials, with good film-forming capability [16,17]. Pectin is a component of the plant cell of the peel of several fruits such as apple and citrus. The properties of pectin are associated with the microstructure that is constituted by poly-α-(1-4)-D-galacturonic acids, known as homogalacturonan [18]. The main structure is made up of at least three polysaccharide domains: homogalacturonan, rhamnogalacturonan-I, and rhamnogalacturonan-II, but homogalacturonan is the major component of pectin polysaccharides [19]. The carboxyl groups of the galacturonic acid units are esterified with methanol and sometimes partially acetyl-esterified units, leading to the formation of high methoxyl pectin, containing more than 50% esterified carboxyl groups, or low methoxyl pectins, containing less than 50% esterified carboxyl groups. Some physical properties, such as the barrier and antimicrobial properties, of pectin films are quite poor. Films based on pure pectin promote microbial growth since pectin is used as a carbon source by fungi and bacteria [20].

Filling pectins with antimicrobials is an interesting way to make such natural materials suitable for food packaging. Essential oils are natural active substances that, apart from their use as flavoring agents, have interesting antimicrobial activity against some bacteria and foodborne pathogens [21,22,23]. Among all the plant-derived essential oils, grapefruit oil (GO), obtained from grapefruit seeds, is known to possess antifungal, antiparasitic, antibacterial, antioxidant, and anticancer properties and exhibits microbial growth inhibition against Gram^+^ and Gram^–^ bacteria [24]. Moreover, to preserve the stability of these substances, the possibility to encapsulate them into nano-containers could be an interesting strategy [25]. In this context, halloysite nanotubes (HNTs) have attracted considerable interest. They are green materials, not hazardous for the environment, and cheaply available in thousands of tons from natural deposits. HNTs consist of two-layered aluminosilicate clay, with the chemical composition Al_2_Si_2_O_5_(OH)_4_·*n*H_2_O. HNTs have a hollow tubular structure similar to kaolin, but their alumosilicate sheets are rolled into tubes, with an external diameter of about 50–80 nm, an internal lumen diameter of 10–15 nm, and a length of about 1000 nm [26,27]. These materials are able to host a wide variety of active molecules in order to release them in a controlled way in specific environments from a polymeric matrix. The combination of pectins and halloysite nanotubes could be the source of novel materials with excellent and unique properties combining the following advantages: macromolecules, derived from renewable resources, nanoparticles, and environmentally friendly [28,29].

This paper reports the preparation and characterization of green composites based on pectins and nano-hybrids of HNTs loaded with GO at different percentages, as antimicrobial agents, for application in food packaging as an active coating. The structural organization and physical properties (thermal, mechanical, barrier) were analyzed and correlated with the nano-hybrid loading. Moreover, analysis of the sustained release of GO was carried out and modeled using a Weibull model. The effectiveness of the fabricated systems was evaluated for strawberry protection used as a fresh food prototype in the active coating field.

## 2. Materials and Methods

### 2.1. Materials

Grapefruit seed oil (GO), 100% pure-cold pressed, was purchased from Organic Herbal Essence. The main components, evaluated by a GC-MS (Thermo Fischer Scientific, Waltham, MA, USA), are: lauric acid 0.68% *w*/*w*, tetradecanoic acid 1.56% *w*/*w*, palmitic acid 36.17% *w*/*w*, octadecanoic acid 2.38% *w*/*w*, oleic acid 27.81% *w*/*w*, linoleic acid 27.38% *w*/*w*, linolenic acid 2.51% *w*/*w*, other compounds 1.51% *w*/*w*, most of them having antimicrobial activity [30]. Pectin from apple (P) (CAS: 9000-69-5), glycerol (CAS: 56-81-5), and halloysite nanoclay powder (HNTs in powder form) (CAS: 1332-58-7) were supplied by Sigma Aldrich (Milano, Italy). Ethanol was purchased from Carlo Erba Reagents (Cornaredo, Italy) (CAS: 64-17-5) and used without further purification.

### 2.2. Preparation of HNT-Grapefruit Seed Oil Nano-Hybrids

Grapefruit seed oil was added to halloysite in different amounts in order to obtain nano-hybrids with oil concentrations equal to 20, 30, and 50% *w*/*w*. The HNT-GO mixture was transferred into a vacuum jar connected to a vacuum pump and kept under vacuum for 30 min in order to remove air from the inner space of the HNTs; then air was added slowly until atmospheric pressure was reached. In this way, the grapefruit seed oil was loaded into HNTs by capillary force. This procedure was repeated three times to increase the loading efficiency. The nano-hybrids obtained were labelled as H*_x_*G*_y_*, where *x* and *y* are the amount (wt%) of HNTs and GO, respectively (Table 1).

### 2.3. Preparation of Nano-Hybrid/Pectin Composites

Nano-hybrid/pectin composites were prepared by dissolving pectin (0.034 g/mL) in distilled water. Glycerol was added as a plasticizer (3 wt%), and the solution was stirred at 80 °C for 1 h. After solubilization, the nano-hybrid H*_x_*G*_y_* (5 wt% on a pectin basis) was added to pectin-glycerol solution, and the mixture was stirred for a further 30 min at 80 °C. In order to allow the best mixing, the solution was milled by high-energy ball milling (HEBM-Retsch-PM 100, Pedrengo, Italy) for 1 h at 350 rpm using five zirconium oxide spheres as a grinding medium. A different solution was prepared for each nano-hybrid composition. The mixtures were then poured into Petri dishes, and the casting process was carried out at room temperature. The obtained nano-hybrid/pectin films were coded as P-H*_x_*G*_y_*, where *x* and *y* are the amount (wt%) of HNTs and GO, respectively. Samples of pure pectin were also prepared using the same experimental procedure. Grapefruit seed oil/pectin films were prepared by adding the required quantity of neat GO to pectin solution under stirring at 80 °C for 1 h and then casted at room temperature. They were labelled as P-G*_y_*, where *y* is the same amount of grapefruit seed oil present in the composites with halloysite. This means that, for example, the corresponding P-G_20_ sample of the composite P-H_80_G_20_ (where P, 95 wt% and H_80_G_20_, 5 wt%) had an amount of GO equal to 1 wt% (Table 1). In order to confirm the encapsulation of the oil into the nanotubes, the release kinetics of P-H*_x_*G*_y_* and the reference P-G*_y_* samples were compared.

### 2.4. Methods

Thermogravimetric analysis (TGA) was utilized for detecting the thermal degradation process that is related to the mass loss of the specimen as a function of rising temperature. The analysis was performed using a Mettler TC-10 thermobalance (Columbus, OH, USA). The test was carried out in a temperature range of 30–700 °C with a heating rate of 10 °C/min under air atmosphere.

Attenuated total reflection (ATR) infrared spectra were recorded by a Bruker (Billerica, MA, USA) spectrometer, model Vertex 70. The incidence angle of the radiation on the ATR crystals was 45°. Analyses were performed by acquiring the background in air and accumulating 32 spectra for each measurement at a resolution of 4 cm^−1^.

X-ray diffraction (XRD) patterns were collected by a Philips-X’Pert MPD X-ray diffractometer (The Netherlands), operating at 40 kV and 40 mA, in the range of 2*θ* = 5–30°, with a step size of 005° and step scan of 10 s, equipped with a Cu sealed tube using Kα radiation (*λ* = 1.54056Å). The data were analyzed using X′ Pert Quantify software.

Mechanical properties were evaluated using a dynamometric apparatus INSTRON 5967 (Norwood, MA, USA) with a cell load of 1 kN. The experiments were conducted on pectin-based films at room temperature in tensile mode with a deformation rate of 1 mm/min. The initial length of the samples was about 20 mm, and data were averaged for five samples.

Transparency of the films was evaluated using an ultra-violet spectrophotometer UV-2401 PC Shimadzu (Kyoto, Japan). The film samples (area 4 cm^2^) were placed into the spectrophotometer cell, and the light transmission was evaluated in the UV-Vis range (200–800 nm). The transparency was determined by evaluating the transparency index (*Tr*) using Equation (1) [31]:

(1) $$\mathrm{Transparency}\;\left(Tr\right)=\frac{Abs_{600}}{x},$$

where *Abs*_600_ is the absorbance at 600 nm and *x* is the film thickness (mm). The lower the transparency index, the higher the transparency.

Barrier properties of water vapour were evaluated through a Dynamic Vapor Sorption (DVS) automated multi-vapour gravimetric sorption analyser using dry nitrogen as the carrier gas. The temperature was fixed to 30 °C. Samples were exposed to increasing water vapour pressures, obtaining different water activities *a_w_* = *P/P*_0_ (from *a_w_* = 0.2 to *a_w_* = 0.8), where *P* is the partial pressure in the gravimetric chamber, and *P*_0_ is the saturation water pressure at the experimental temperature. The adsorbed water mass was measured by a microbalance and recorded as a function of time. The sorption parameter was estimated by evaluating the derivative of the equilibrium moisture content (*M*) with respect to the partial pressure (*P*), according to Equation (2):

(2) $$S=\frac{dM}{dP}$$

The estimation of the diffusion coefficient was obtained through the approximated form of Fick’s second law, represented by Equation (3):

(3) $$\frac{m}{m_{eq}}=\frac{4}{d}\sqrt{\frac{Dt}{\pi }}$$

where *m_eq_* is the mass moisture when saturation is reached, *d* is the characteristic length of the sample (cm), and *D* (cm^2^/s) is the diffusion coefficient.

The water permeability coefficient was then obtained from the product of sorption and diffusion (Equation (4)):

(4) $${P=S\;\times \;D}_{0}$$

The grapefruit seed oil composition was determined and reported in a previous paper [32]. The release kinetics of linoleic acid from nanocomposites, the major component of the grapefruit oil, were followed using a Shimadzu UV-2401 PC spectrophotometer. The tests were performed using 4 cm^2^ rectangular specimens with the same thickness (≈300 μm) placed in 25 mL ethanol and stirred at 100 rpm in an orbital shaker (VDRL MOD. 711+, Asal S.r.l., Cernusco sul Naviglio, Italy). The release medium was withdrawn at fixed time intervals and replaced with fresh medium. The absorption band under investigation was located at 208 nm. The concentration at each fixed time was evaluated using a calibration curve (Absorbance = 0.0088 × *c* (mg/L)). Then, the release drug amount was easily calculated by multiplying the concentration *c* (mg/L) by the solution volume (25 mL) and was normalized with respect to theoretical initial loaded amount of the drug (*c*_0_).

In order to analyze the multi-stage release kinetics of linoleic acid, a modified Weibull method (Equation (5)) was applied [33]:

(5) $$\frac{M}{M_{0}}=\theta \left(1-{\exp\;}^{-\frac{1}{A_{1}}{\times t}^{b_{1}}}\right)+\left(1\;-\;\theta \right)*\left(1-{\exp\;}^{-\frac{1}{A_{2}}\times {\left(t-t_{m}\right)}^{b_{2}}}\right)$$

where *M* represents the amount of drug dissolved as a function of time *t*, *M*_0_ is the theoretical initial loaded amount of the drug. The first contribution with weight *θ* represents the contribution of the diffusion-controlled mechanism while the second contribution (1 *− θ*) represents the contribution of the slow release due to polymeric chain relaxation. Factors *A*_1_ and *A*_2_ account for the time dependence, parameters *b*_1_ and *b*_2_ are related to the drug release mechanism, and parameter *t_m_* accounts for the maximum cumulative drug release time of stage 1.

Coating tests on fresh strawberries were carried out. Fruits were thoroughly washed and wiped with cellulose sheets. The strawberries were then dipped in the three coating formulations for 2 min, and then the excess coating materials were left to drip off. Paper-based trays were used for fruit packing and storage. The coated fruits were stored at room temperature for 10 days under a relative humidity (*RH*) of 60%. Uncoated strawberries were used as controls and were stored under the same conditions.

The statistical significance of the obtained data was assessed by performing one-way ANOVA. Tukey’s post hoc method was carried out for assessing significant differences between means (*p* < 0.05). The statistical comparisons were performed using GraphPad Prism 9 software.

## 3. Results

### 3.1. Characterization of HNT-Grapefruit Seed Oil Nano-Hybrids

**Figure 1:**
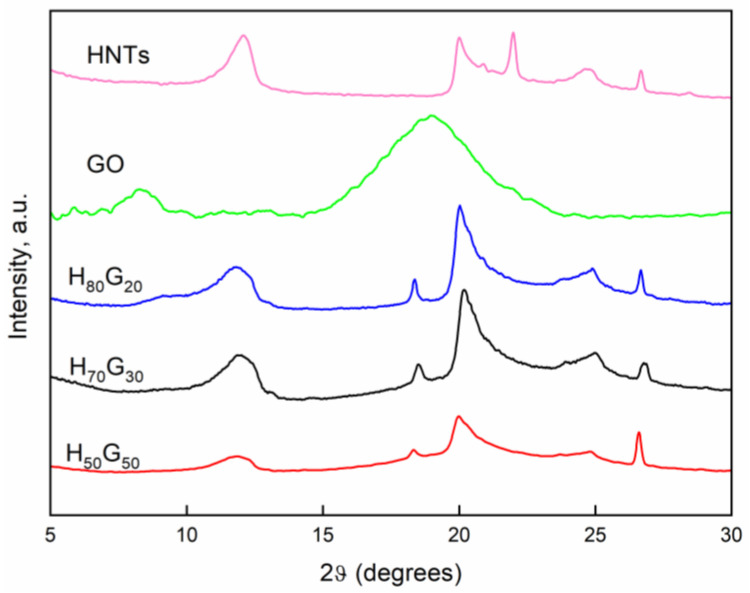
XRD pattern of halloysite nanotubes (HNTs), grapefruit seed oil (GO), and nano-hybrids (H*_x_*G*_y_*).

The XRD patterns of the produced nano-hybrids (H*_x_*G*_y_* fillers, *x* mass percentage of HNTs, *y* mass percentage of grapefruit seed oil) are reported in Figure 1. The spectra of pristine halloysite and grapefruit seed oil are reported.

X-ray diffraction analysis revealed that the halloysite nanoclay (HNT) was largely present in nano-tubular form as suggested from the characteristic peak at 2*θ* = 20.0° (*d* = 4.43 Å, using Bragg’s Law) related to the (100) crystalline plane [34]. Moreover, the nanotubes were in a dehydrated state as evident from the peak of 2*θ* at 12.09°, which translates into a (001) basal spacing of the mesoporous structure in nanotubes, *d* = 7.31 Å, and the absence of a sharp peak of 2*θ* at ~8.76°, which is otherwise suggestive of the hydrated status of the nanotubes [35]. Thus, the halloysite used in this study was fully dehydrated. Further confirmation of the dehydrated state was the presence of the (002) basal reflection at 24.6° 2*θ* (equivalent to *d* = 3.61 Å).

The XRD reflection peak of H*_x_*G*_y_* fillers of 2*θ* at around 12° broadened with the increasing amount of GO and very slightly shifted to lower 2*θ* values (Table 2), indicating a minimal increase in the interlayer distance in comparison with that of the unmodified HNTs probably due to GO encapsulation [36].

**Figure 2:**
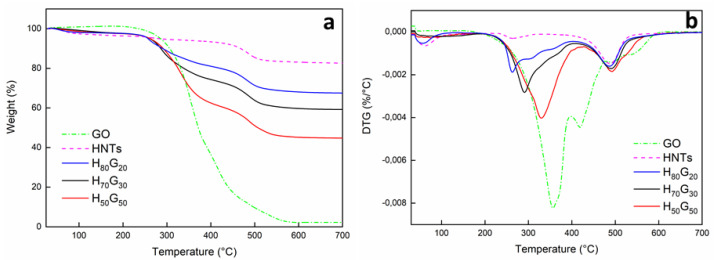
TGA (**a**) and DTG (**b**) curves of HNTs, GO, and nanohybrids.

Thermogravimetric analysis allowed the determination of material thermal stability. Figure 2 shows the thermogravimetric curves (a) and DTG curves (b) of fabricated nano-hybrids. TG and DTG curves of unmodified HNTs and GO are reported for comparison.

The mass loss profiles of halloysite nanotubes were characterized by an only mass-loss stage at around 500 °C, revealing a mass change of 17.4% corresponding to the dehydroxylation of aluminol groups [37].

Decomposition of grapefruit seed oil involved a wide temperature range (from 280 up to 590 °C), which could be associated with the degradation of saturated and unsaturated fatty acids in the oil.

The TGA curves of the fabricated nanohybrids were very similar and were depicted by the characteristic degradation steps of both HNTs and GO with increasing thermal stability as the HNT content increased. The amount of inorganic component in the samples H*_x_*G*_y_*, evaluated as oxides at 700 °C, are in good agreement with the nanohybrids composition (Table 3). The main decomposition step of GO appeared to be shifted towards lower temperatures (*Td*_1*(DTG)*_) after the encapsulation into clay nanotubes, and a noticeable reduction of the decomposition rate could be observed. The decomposition temperatures of HNTs (*Td*_2*(DTG)*_) remained almost constant at around 490 °C for all nano-hybrids.

### 3.2. Characterization of Nano-Hybrid/Pectin Composites

**Figure 3:**
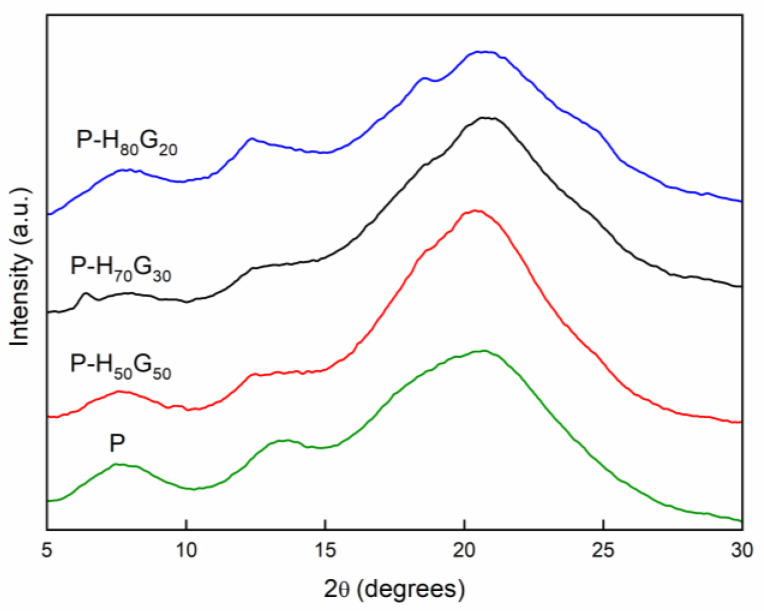
XRD pattern of pectin and pectin composites (P-H*_x_*G*_y_*).

Figure 3 reports the XRD spectra of pectin film and the nano-hybrid/pectin composites. The plot of pectin shows amorphous peaks centred around 7.6°, 13.4°, and 20.5° of 2*θ*. Upon incorporation of nano-hybrids into the pectin solution, the composite films presented the main peaks around 7.7°, 12.4°, 18.5°, and 20.6° of 2*θ* as superposition of the pectin and H*_x_*G*_y_* diffraction features. Polymer composites resulted as physical mixtures of the constituent components.

**Figure 4:**
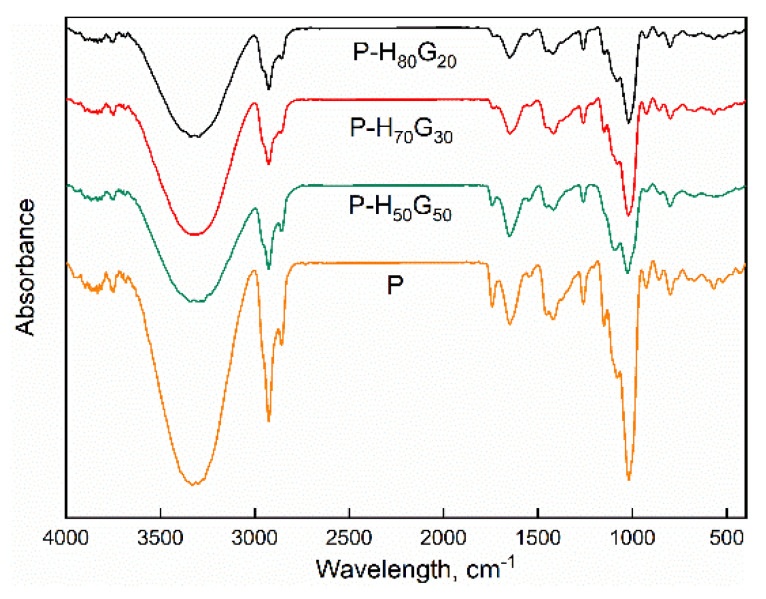
ATR spectra of pectin and pectin nanocomposites.

ATR spectra were collected for all samples and are depicted in Figure 4.

The appearance of a very broad band between 3000 and 3600 cm^−1^ is attributed to the OH bond vibrations of the secondary alcohol of pectin [38], while the characteristic bands of aliphatic hydrocarbons related to the stretching, bending, and rocking vibrations of CH groups are visible at 2930 and 2860 cm^−1^ [39]. The vibration peak at 1645 cm^−1^ can be mainly attributed to bound water. The region 1900–1000 cm^−1^ displays the peaks of C=O, symmetric and asymmetric COO–, and COC stretching, characteristic of vibrational peaks of pectin [40,41]. Moreover, the small peak shoulder at 1373 cm^−1^ belongs to OH groups of carboxylic acids of pectin, the band at 1454 cm^−1^ belongs to CH_2_ bending vibration [42], while the peak at 1017 cm^−1^ suggests –CH–O–CH– stretching. The characteristic bands of aliphatic hydrocarbons in the essential oil are present at 3000–2800, 1465–1377, and 720 cm^−1^, respectively [43]. C–H out-of-plan bending of fatty acids is visible at 970 cm^−1^. In the FTIR spectra of P-H*_x_*G*_y_*, characteristic bands of HNTs are present [44]: the band at 3200–3500 cm^−1^ (O−H stretching vibrations attributed to crystal water), the band at 910 cm^−1^ (Al−OH bending vibration), bands at 1000–1100 cm^−1^ (Si−O stretching vibration), and 450–550 cm^−1^ (Si−O bending vibration and Al–O–Si) [45,46].

**Figure 5:**
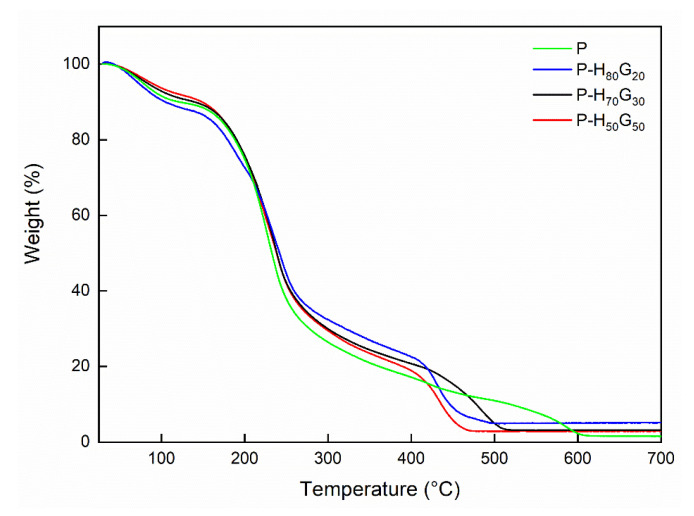
TGA curves of pectin and pectin composites.

Thermo-oxidative degradation of pectins is known to be a series of complex phenomena. Pectin and pectin composites display a three-step thermal degradation behaviour as shown in Figure 5. The first step up to ~150 °C is related to the evaporation of water molecules. The second one from 150 to around 250 °C is related to the pyrolytic decomposition due to the primary and secondary decarboxylation involving the acid side group and a carbon in the ring [47]. The last step corresponds to the start of the oxidation process. No appreciable differences were evident among the pectin samples (*p* < 0.05). Regarding the last step, the characteristic decomposition peak shifted at lower temperatures after the introduction of hybrid filler. HNTs are supposed to act as flame retardants for polymers [48]. Therefore, the decomposition of volatile products as well as the oxidation of carbon residues could have a catalytic effect, promoting pectin oxidation [25].

Mechanical performances were evaluated by carrying out stress–strain tests. The parameters of interest are reported in Table 4:

An enhancement of the elastic modulus of pectin composites in compliance with the amount of HNTs (26%, 49%, and 67% for P-H_50_G_50_, P-H_70_G_30_, and P-H_80_G_20_, respectively) was observed. This phenomenon can be related to the reinforcing effect of HNTs via the formation of hydrogen bonds [25]. The strength at break point increased after the loading of the hybrid filler, with an enhancement of 26%, 42%, and 48% for P-H_50_G_50_, P-H_70_G_30_, and P-H_80_G_20_, respectively. These results confirm the good dispersion of hybrid filler (even varying the ratio HNTs/GO) and the absence of cluster formation, which would contribute to worsen the mechanical performances. The elongation at break point appeared to increase for pectin composites due to the plasticizing effect of glycerol. Moreover, no noticeable differences in stress at break point were evident between the loaded pectin composites.

**Figure 6:**
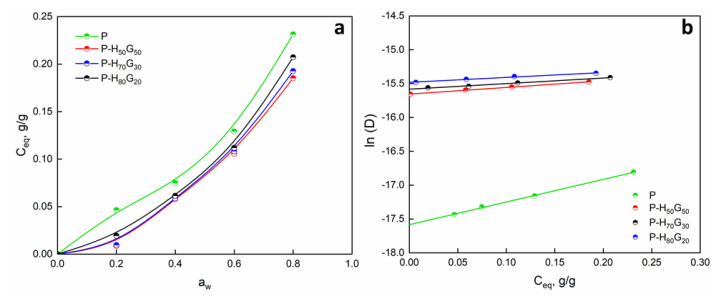
(**a**) Sorption isotherm and (**b**) diffusivity vs. equilibrium concentration of pectin and pectin composites.

Barrier properties were studied, and sorption, diffusion, and permeability parameters were calculated (Table 5). Sorption isotherms and diffusion coefficients are reported in Figure 6.

The presence of domains of inorganic phases contributed to make the systems more complicated. The sorption curve of pure pectin followed a typical dual sorption behaviour: at low activity (*a_w_* < 0.2), an increase in water adsorption was followed by a linear behaviour, indicating the occurring of the dissolution process and the sorption of the water molecules on preferential sites with finite adsorption capacity [17]. At higher *a_w_*, water induced polymer plasticization, and the curve turned into an exponential trend. The sorption isotherm of pectin composites followed the typical type III isotherm according to Brunauer–Emmett–Teller (BET) classification [49]. The introduction of hybrid filler contributed to decrease the amount of sorbed water. This effect can be associated with the reduction of active polar sites after the introduction of HNTs as well as the hydrophobicity of GO. It suggests that the polar group of the pectin chains are less free for the sorption of the water molecules since they are hindered by the clay. However, at high *a_w_*, the matrix structure was plasticized by the entering water molecules, which induced higher sorption [50]. No noticeable differences were observed among the filler pectin films. This was proved by the similar sorption coefficients (*S*) of the pectin composites. As shown in Figure 6b (diffusion coefficient versus equilibrium water concentration), the diffusion coefficient increased for pectin composites compared to neat pectin. The *D* parameter increased linearly with *C_eq_*, but no differences were evident among pectin composites. The improvement of the diffusivity of the composites versus neat pectin film can be ascribed to the effect of HNTs/GO filler, which could create local disconnection points inside the polymeric matrix, contributing to the increase in porosity and an increase in the diffusion. The extrapolation at *C_eq_* = 0 of the diffusion linear trend allowed us to extrapolated the *D_0_* coefficient (Table 5), which provides information concerning the structure of the matrix (free volume and tortuosity of the path) and thus the morphological texture.

The permeability *P* of the composites was evaluated as the product of *D* × 10^7^ (cm^2^/s) and *S* (g/g∗atm^−1^) according to Equation (4). An increase in the permeability value was evident for all pectin composites without significant differences among them. This was largely dominated by the diffusion phenomenon, as shown by the respective values of sorption and diffusion reported in Table 5. It is important to emphasize that such permeability is an ideal value, which is valid in a low vapor concentration range.

**Figure 7:**
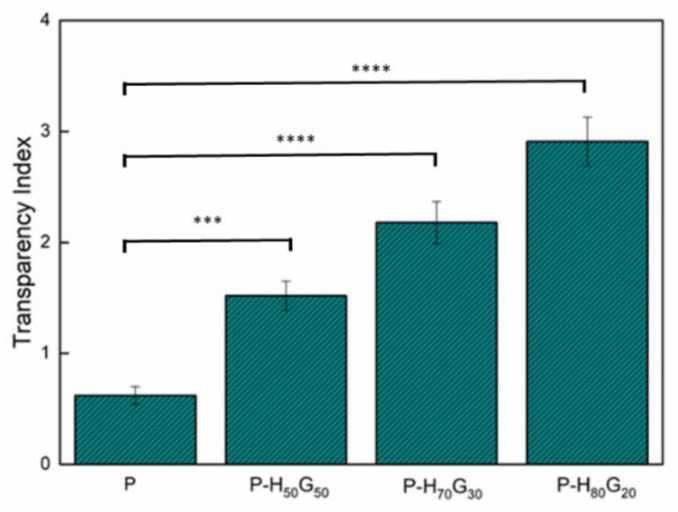
Transparency index of neat pectin and pectin composites. Statistically significant differences: *** *p* < 0.001 and **** *p* < 0.0001.

Transparency (*Tr*) is a fundamental parameter of bio-based films for application in food-packaging fields [51]. Composite pectin films containing hybrid filler were homogeneous with a dark yellowish colour. Since the lower the transparency index, the higher the transparency, pectin film was the most transparent sample. The loading of hybrid filler (H*_x_*G*_y_*) into pectin slightly reduced the transparency of the films. The transparency indices of neat pectin and its composites are reported in Figure 7. The increase in the filler concentration led to a reduction of the free volume. Moreover, oil molecules around the HNT surface enhanced the light scattering and, consequently, the opacity of the films. This behaviour could be associated with the change in the film refractive index induced by the filler presence [52,53].

Therefore, the presence of the hybrid filler effectively acted as light absorber, reducing the transmission of visible light while still keeping good transmittance of the radiation, allowing clear inspection of the food content inside the packaging.

**Figure 8:**
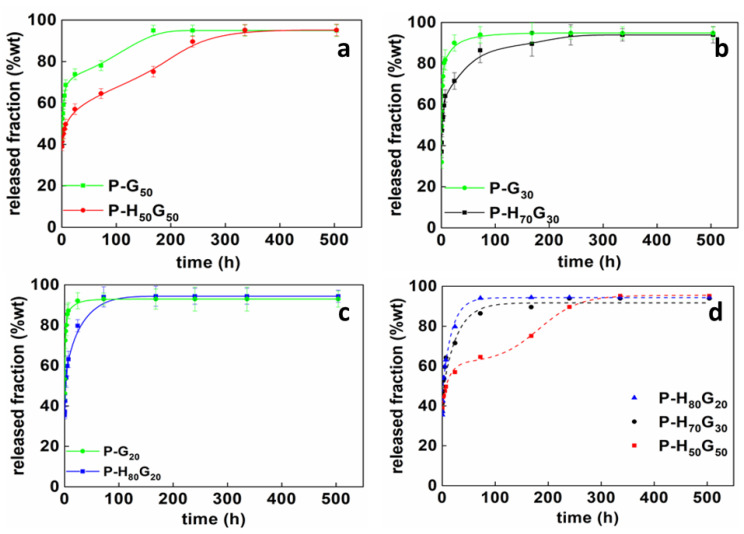
Release profiles of (**a**) P-H_50_G_50_, (**b**) P-H_70_G_30_, and (**c**) P-H_80_G_20_ compared to HNT-free pectin composites and (**d**) hybrid pectin composites.

Figure 8 shows the release profiles of the hybrid composites (P-H*_x_*G*_y_*) compared to free dispersed GO in the pectin matrix (P-G*_y_*) as well as the comparison between all pectin-based composites with different filler compositions.

All samples showed a sustained release behavior, although an initial rapid release of linoleic acid was found within the first 24 h followed by a second stage that showed a slow active molecule release, which was attributed mainly to the diffusion or permeation of the molecules through the polymer matrix towards the release medium.

In particular, the release kinetics of P-G_50_ and P-H_50_G_50_ showed an initial burst release with 50 wt% and 70 wt% linoleic acid-released fraction, respectively, and a plateau regime was reached after 7 and 14 days. For samples P-G_30_ and P-H_70_G_30_, the burst phenomenon involved the linoleic acid release of 60 wt% and 80 wt%, respectively, while the equilibrium state was reached after 3 and 10 days. Finally, the first fast release stage for P-G_20_ and P-H_80_G_20_ regarded the 55 wt% and 85 wt% released fraction, respectively, and the second equilibrium stage occurred after 1 and 3 days. The initial fast release (up to 24 h) is related to the diffusion of free oil molecules and molecules attached to the HNT surface (Figure S1) while the encapsulation into a nanometric system proved the noticeable slowing of the release rate. To better investigate the effect of the hybrid filler and its different composition (ratio of HNTs/GO), Figure 8 reports the comparison between the produced composites. It is worthwhile highlighting that only the composite P-H_50_G_50_ showed a multi-step release profile. This effect could be related to the relaxation of polymeric chains induced by the high amount of GO in P-H_50_G_50_. In order to analyze the multi-stage release kinetics of hybrid composites, a modified Weibull method was applied (Table 6).

The contribution of the diffusion-controlled mechanism (*θ*) for the composite P-H_50_G_50_ was about 66% while the contribution of the slow release due to polymeric chains relaxation (1 − *θ*) was 34%, with an inflection point reached at about 130 h; otherwise, no relaxation occurred in the composites P-H_70_G_30_ and P-H_80_G_20_.

**Figure 9:**
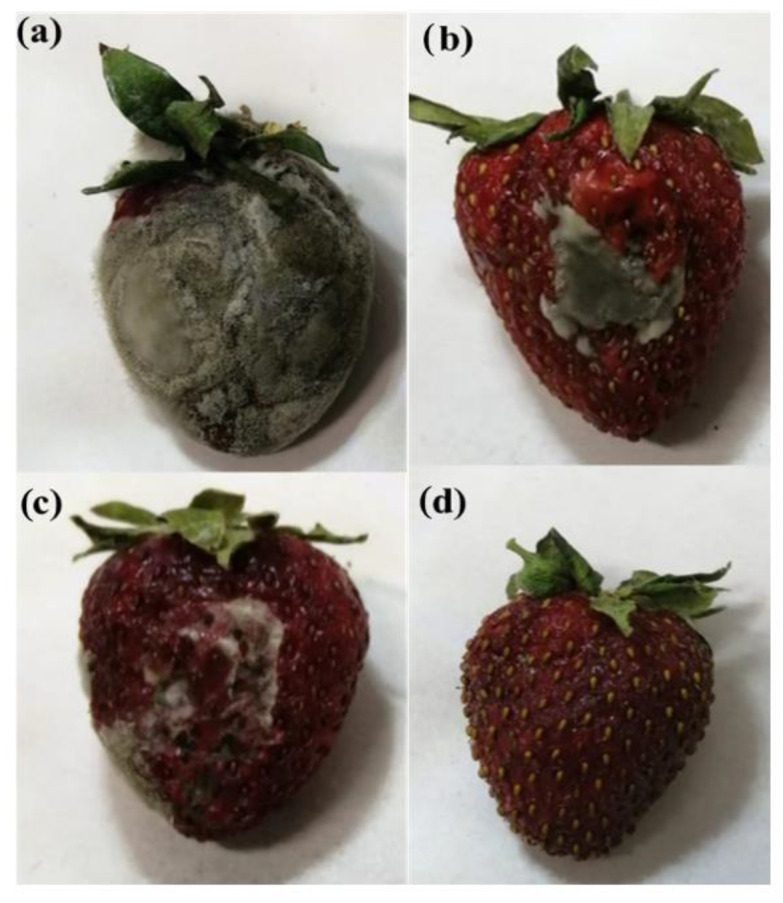
Fresh strawberry: (**a**) uncoated; (**b**) coated with P-H_80_G_20_; (**c**) coated with P-H_70_G_30_, and (**d**) coated with P-H_50_G_50_ after 10 days of storage at room temperature, *RH* = 60%.

Preliminary studies of the effect of antimicrobial GO on coated and uncoated fruits were carried out by simply visually following the formation of mould. Figure 9 shows pictures of uncoated and coated strawberries with different coating formulations after 10 days of storage at room temperature, *RH* = 60%. The active pectin-based coating was proven to prevent mould formation and to extend the time of storage of such fruits. The uncoated fruit appeared to be completely covered by mould after two days, with a wrinkled and damaged appearance (Figure 9a). Preliminary microbiological spoilage assessments were visible in comparison with the coated fruits. Mould started to spread on coated fruits after 3–4 days. The appearance of mould was quite evident for samples P-H_80_G_20_ (Figure 9b) and P-H_70_G_30_ (Figure 9c), while a noticeable mould prevention effect was ensured in sample with the highest GO amount, P-H_50_G_50_ (Figure 9c), whose covering fully protected the strawberry from degradation. The results seem to be affected by GO content. Indeed, the ester groups of pectin chains are supposed to be good acceptors of hydrogen bonds trapping water molecules in proximity to the polymer [54]. An increase in the GO amount in the hybrid filler allowed better preservation of the fruit texture and appearance, proving and corroborating its known effect as an antimicrobial agent. These findings suggest that the combination of the pectin coating and antimicrobial properties of GO was efficient in the shielding of gas flow as well as the extension of the shelf-life of strawberries.

## 4. Conclusions

Novel bio-coatings based on pectin and grapefruit seed oil, used as an antimicrobial agent, encapsulated into halloysite nanotubes, were prepared and tested. Different ratios of GO/HNTs were used to produce bio-nanocomposites. The hybrid filler was characterized in terms of thermal stability, which proved the effect of HNTs as a flame retardant. Then, the physical properties of the pectin composites were studied. TGA results showed that the degradation of the pectin matrix was not influenced by the filler. With respect to the mechanical performance, the presence of the hybrid filler led to an enhancement of the elastic modulus and stress at break point parameters due to the reinforcing effect of HNTs. Sorption isotherms showed a reduction in the amount of adsorbed water, while an increase in the diffusion coefficient was observed probably due to the formation of local disconnection points. Transparency index evaluation proved the slight increase in opacity due to the presence of HNTs and essential oil, which could cause a light-scattering phenomenon although without compromising a good visual inspection of the foodstuff. Coating tests on fresh strawberries showed significant extension of the preservation time compared to uncoated ones. This result indicates the potential application of the prepared formulations in the food packaging field and opens new perspectives in using pectin-based composites as bio-coating agents, either directly in contact with selected foods or in polymer-based multilayer packaging systems. Tests are in progress to test the bacterial inhibitory growth of pectin composites in order to evaluate a threshold antimicrobial value of the inhibitory concentration of nano-fillers.

## Figures and Tables

**Table 1:** Acronyms and composition of nano-hybrid/pectin composites (P-H*_x_*G*_y_*) and grapefruit seed oil/pectin composites (P-G*_y_*).

Sample	P (wt%)	H*_x_*G*_y_* (wt%)	GO (wt%)
P-H_80_G_20_	95	5	-
P-H_70_G_30_	95	5	-
P-H_50_G_50_ P-G_20_ P-G_30_ P-G_50_	95 99 98.5 97.5	5 - - -	- 1 1.5 2.5

**Table 2:** Diffraction peaks (2*θ*) and corresponding interlayer distance (*d*) for neat HNTs and all nano-hybrids.

Sample	(001)		(100)	
	2*θ*	*d* (Å)	2*θ*	*d* (Å)
HNTs	12.09	7.31	20.0	4.43
H_80_G_20_	11.82	7.48	20.02	4.43
H_70_G_30_	11.87	7.45	20.18	4.40
H_50_G_50_	11.87	7.45	19.97	4.44

**Table 3:** TG-DTG analysis of tested materials: HNTs and GO content, mass loss, and decomposition temperature.

Sample	HNTs Content (% *w*/*w*)	GO Content (% *w*/*w*)	Mass Loss (% *w*/*w*)	*Td*_1*(DTG)*_ (°C)	*Td*_2*(DTG)*_ (°C)
HNTs	100	0	82.6	-	488
GO	0	100	2.2	356	-
H_80_G_20_	80	20	67.5	262	487
H_70_G_30_	70	30	59.2	291	491
H_50_G_50_	50	50	44.8	330	492

*Td*_1*(DTG)*_ and *Td*_2*(DTG)*_ represent the DTG temperature of the peak (°C) corresponding to the decomposition of GO and HNTs, respectively.

**Table 4:** Mechanical parameters of pectin and pectin composites.

Parameter	P	P-H_50_G_50_	P-H_70_G_30_	P-H_80_G_20_
E [MPa]	32.53 ± 5.22 ^c^	47.87 ± 6.27 ^bc^	64.25 ± 6.95 ^b^	98.54 ± 6.03 ^a^
σ_break_ [MPa]	1.78 ± 0.15 ^c^	2.41 ± 0.19 ^b^	3.05 ± 0.21 ^a^	3.45 ± 0.26 ^a^
ε_break_ [%]	29.68 ± 3.12 ^b^	39.95 ± 3.78 ^a^	41.32 ± 4.05 ^a^	37.12 ± 3.89 ^ab^

E: Elastic modulus; σ_break_: stress at breaking; ε_break_: deformation at breaking. For each composite, different superscript letters in the same row indicate that the mean values are significantly different (*p* ≤ 0.05).

**Table 5:** Sorption, diffusion, and permeability values of pectin and pectin composites.

Parameter	P	P-H_50_G_50_	P-H_70_G_30_	P-H_80_G_20_
*S* (g/g∗atm^−1^)	4.40 ± 0.35 ^a^	3.61 ± 0.22 ^ab^	3.51 ± 0.17 ^abc^	3.46 ± 0.26 ^abc^
*D*_0_ × 10^7^ (cm^2^/s)	0.25 ± 0.03 ^b^	1.59 ± 0.19 ^a^	1.81 ± 0.26 ^a^	1.89 ± 0.15 ^a^
*P* × 10^7^ (g/g∗atm^−1^∗cm^2^/s)	1.12 ± 0.08 ^b^	5.74 ± 0.47 ^a^	6.36 ± 0.79 ^a^	6.54 ± 0.52 ^a^

For each composite, different superscript letters in the same row indicate that the mean values are significantly different (*p* ≤ 0.05).

**Table 6:** Weibull’s model parameters obtained after fitting the release data.

Sample	*θ*	*A*_1_ (*h*^*b*1^)	*b*_1_	*A*_2_ (*h*^*b*2^)	*b*_2_	*t_m_ (h)*
P-H_50_G_50_	0.66	1.86	0.14	7.5	0.53	128.6
P-H_70_G_30_	1	1.60	0.24	-	-	-
P-H_80_G_20_	1	2.03	0.35	-	-	-

## Data Availability

Not applicable.

## Supplementary Materials

The following supporting information can be downloaded at: https://www.mdpi.com/article/10.3390/nano12081265/s1, Figure S1: Release profiles of hybrid pectin composites (up to 24 h).
