# Supplementary material for: Fabrication and Characterization of Bio-Nanocomposites Based on Halloysite-Encapsulating Grapefruit Seed Oil in a Pectin Matrix as a Novel Bio-Coating for Strawberry Protection
Source: Nanomaterials (Basel). 2022 Apr 8;12(8):1265. doi: 10.3390/nano12081265 (PMC9025479; doi:10.3390/nano12081265)
Supplement: Supplementary file 1 [file nanomaterials-12-01265-s001.zip › nanomaterials-1654018-supplementary.pdf]

Supporting Information

Fabrication and Characterization of Bio-Nanocomposites Based on Halloysite-Encapsulating Grapefruit Seed Oil in a Pectin Matrix as a Novel Bio-Coating for Strawberry Protection

Gianluca Viscusi [1], Elena Lamberti [1], Francesca D'Amico [1], Loredana Tammaro [2] and Giuliana Gorrasi [1,*]

[1] Department of Industrial Engineering, University of Salerno, via Giovanni Paolo II, 132, 84084 Fisciano (SA), Italy; gviscusi@unisa.it (G.V.); ellamberti@unisa.it (E.L.); f.damico15@studenti.unisa.it (F.D.)

[2] Laboratory of Nanomaterials and Devices (SSPT-PROMAS-NANO), ENEA-C.R. Portici, Piazzale Enrico Fermi 1, 80055 Portici (NA), Italy; loredana.tammaro@enea.it

* Correspondence: ggorrasi@unisa.it

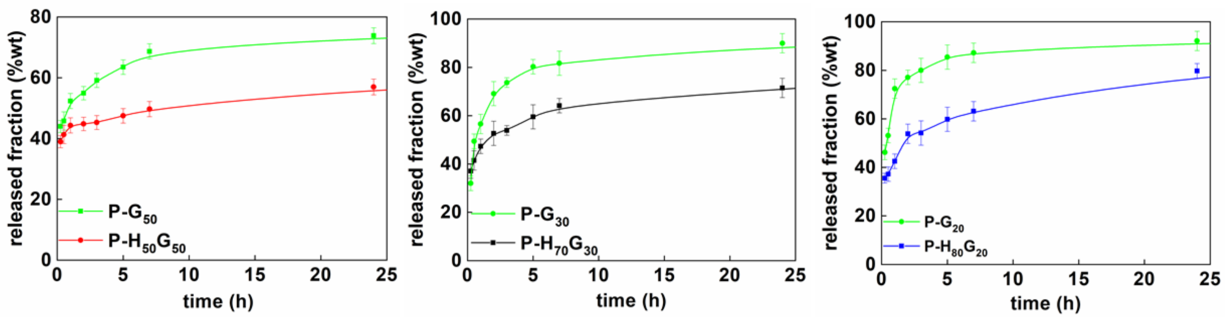

Figure S1. Release profiles of hybrid pectin composites (up to 24 h).
